# Supplementary material for: Study of the Molecular Architectures of 2-(4-Chlorophenyl)-5-(pyrrolidin-1-yl)-2H-1,2,3-triazole-4-carboxylic Acid Using Their Vibrational Spectra, Quantum Chemical Calculations and Molecular Docking with MMP-2 Receptor
Source: Pharmaceutics. 2023 Nov 27;15(12):2686. doi: 10.3390/pharmaceutics15122686 (PMC10747663; doi:10.3390/pharmaceutics15122686)
Supplement: Supplementary file 1 [file pharmaceutics-15-02686-s001.zip › pharmaceutics-2676157-supplementary.pdf]

SUPPLEMENTARY MATERIAL

Study of the Molecular Architectures of 2-(4-Chlorophenyl)-5-(pyrrolidin-1-yl)-2*H*-1,2,3-triazole-4-carboxylic Acid Using Their Vibrational Spectra, Quantum Chemical Calculations and Molecular Docking with MMP-2 Receptor

Mauricio Alcolea Palafox [1,*], Nataliya P. Belskaya [2] and Irena P. Kostova [3,*]

[1] Departamento de Química Física, Facultad de Ciencias Químicas, Universidad Complutense, 28040 Madrid, Spain
[2] Department of Technology for Organic Synthesis, Ural Federal University, 19 Mira Str., 620012 Yekaterinburg, Russia
[3] Department of Chemistry, Faculty of Pharmacy, Medical University of Sofia, 2 Dunav Str., 1000 Sofia, Bulgaria
* Correspondence: alcolea@ucm.es (M.A.P.); irenakostova@yahoo.com (I.P.K.)

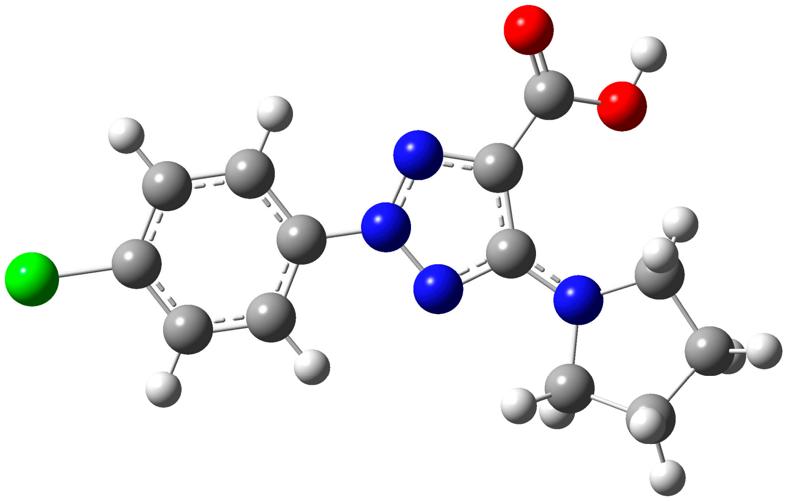

E(B3LYP) = −1332.610258 A.U. (G = −1332.658313 AU)

Figure S1. Optimized structure of **1b** at the B3LYP/6-31G(d,p) level in another conformation.

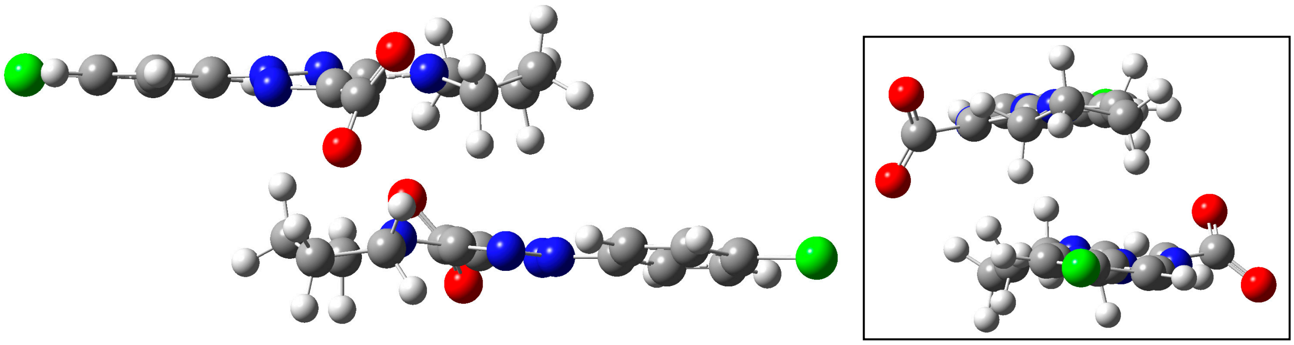

Figure S2. Two views of the optimized dimer *form I* of **2b** at the MP2/6-31G(d,p) level.

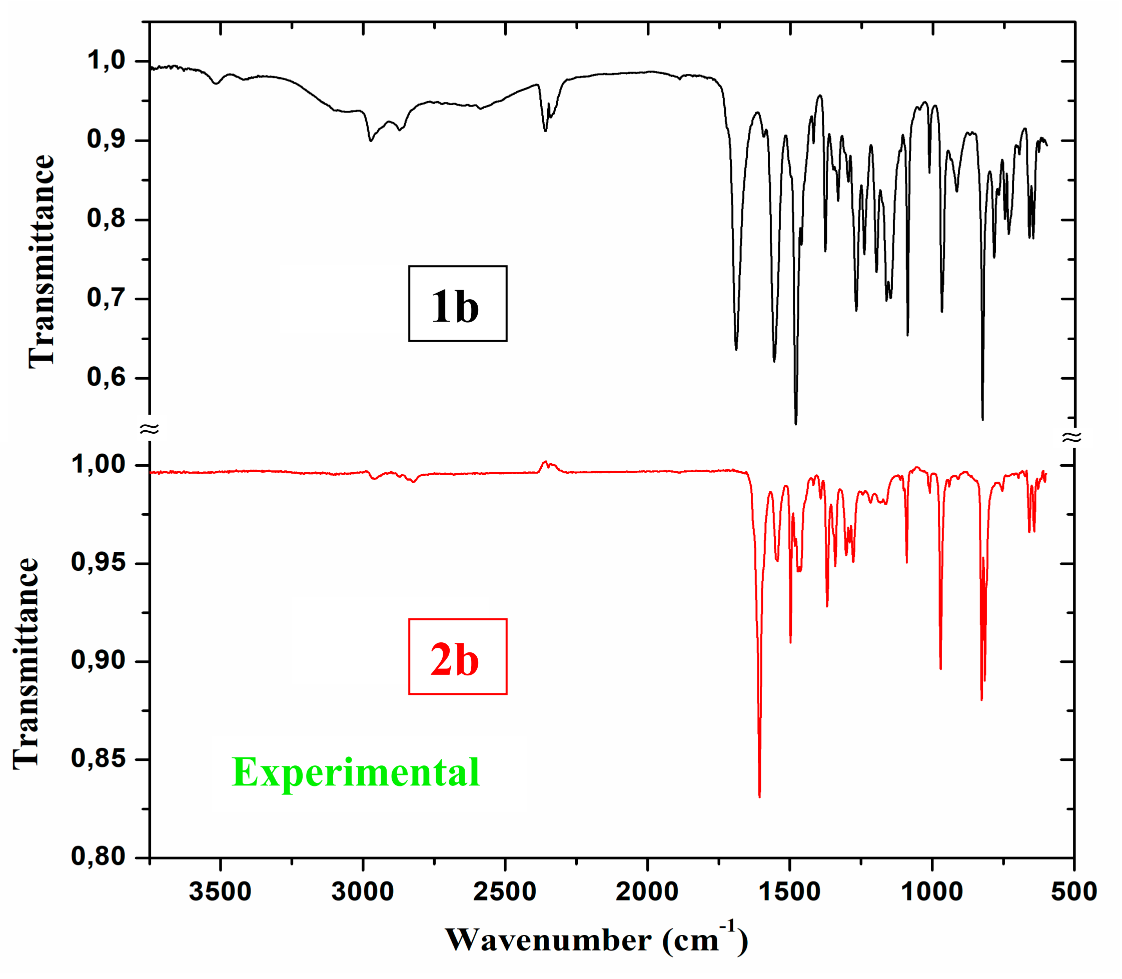

Figure S3. Comparison of the theoretical scaled IR spectra at the B3LYP/6-31G(d,p) level with the experimental ones of **1b** and **2b** molecules in the 3750-500 cm^{-1} range.

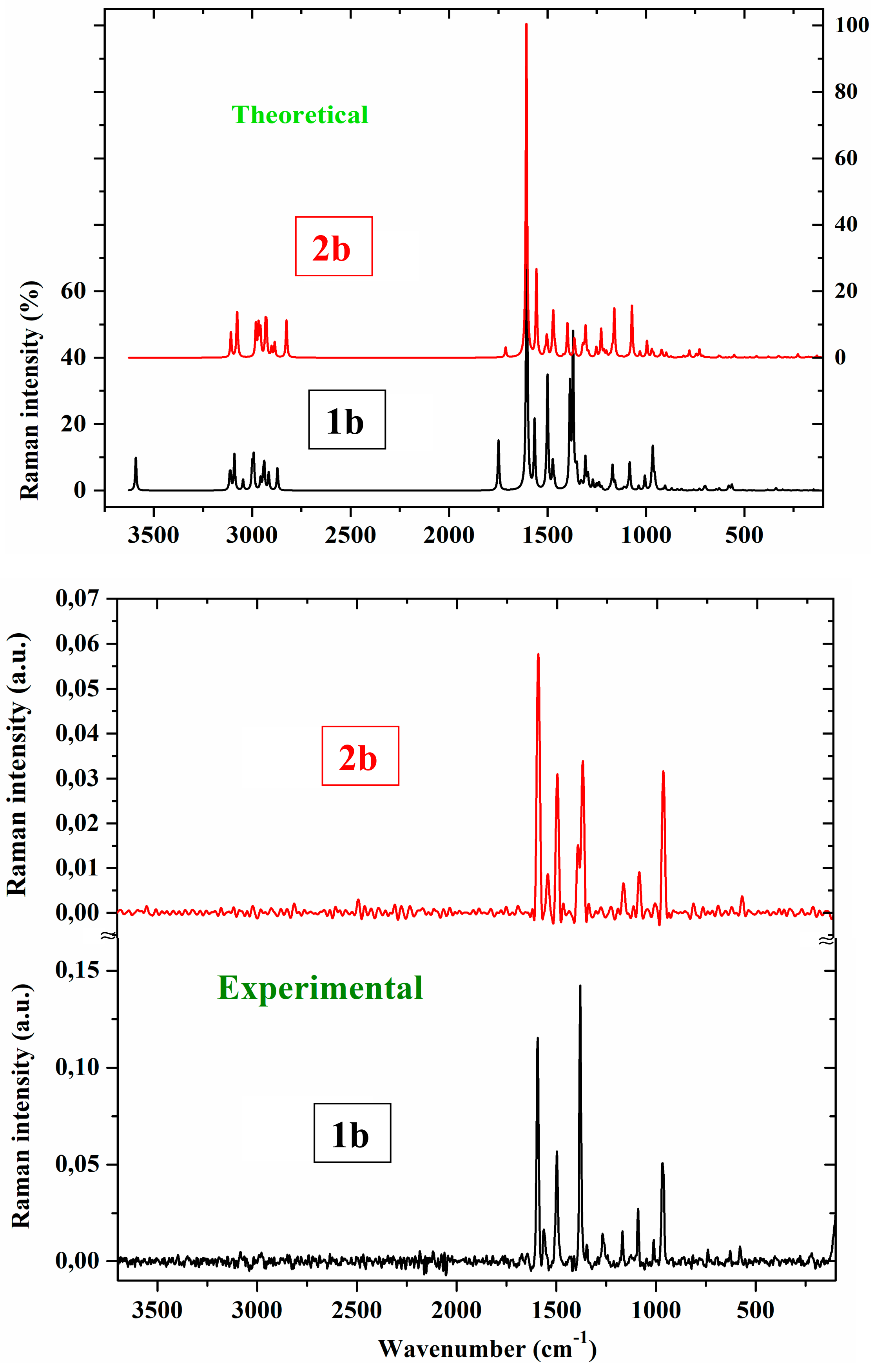

Figure S4. Comparison of the theoretical scaled Raman spectra at the B3LYP/6-31G(d,p) level with the experimental ones in the 3750-100 cm^{-1} range of **1b** and **2b** molecules.

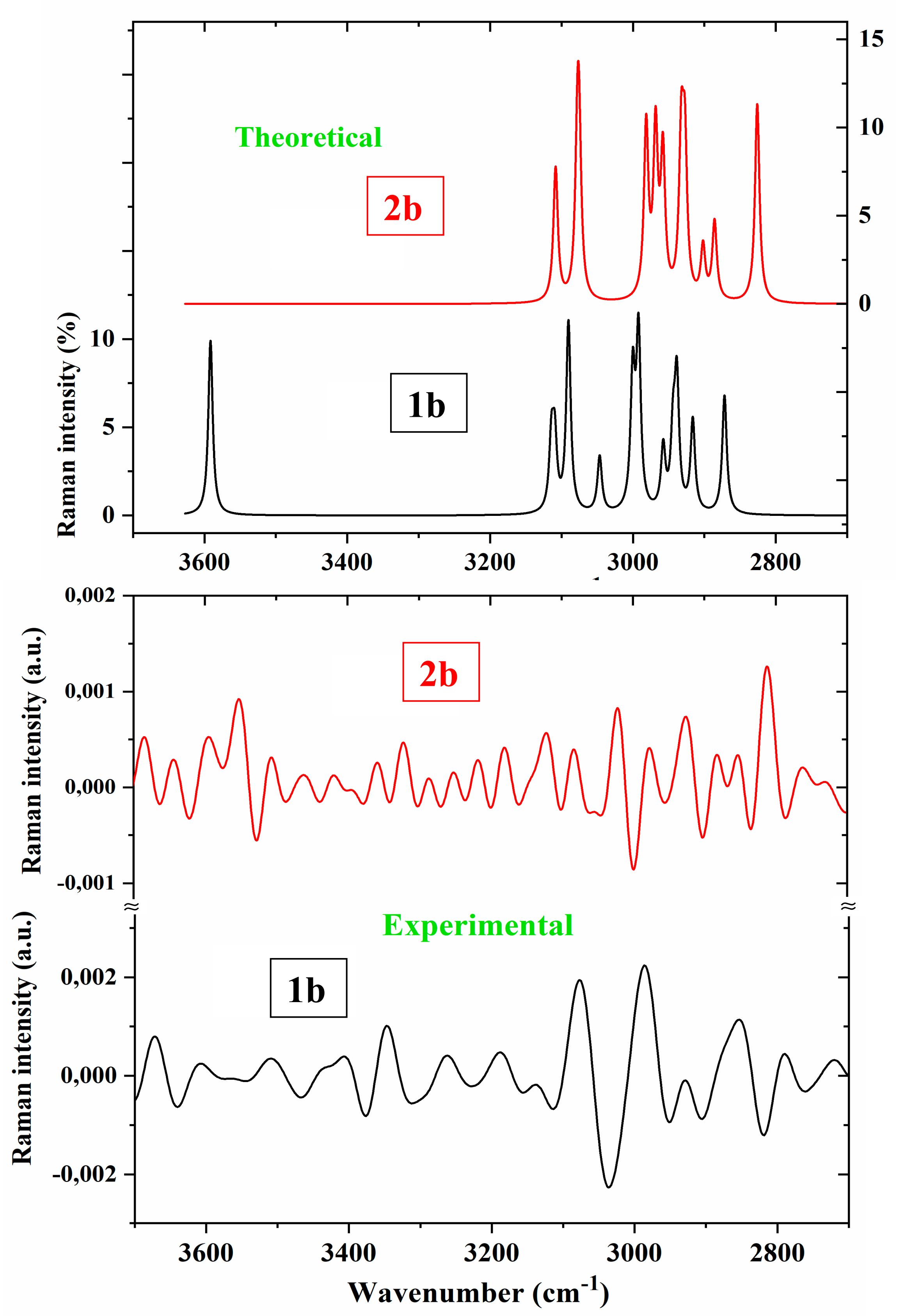

Figure S5. Comparison of the theoretical scaled Raman spectra at the B3LYP/6-31G(d,p) level with the experimental ones of **1b** and **2b** molecules in the 3700-2700 cm^{-1} range.

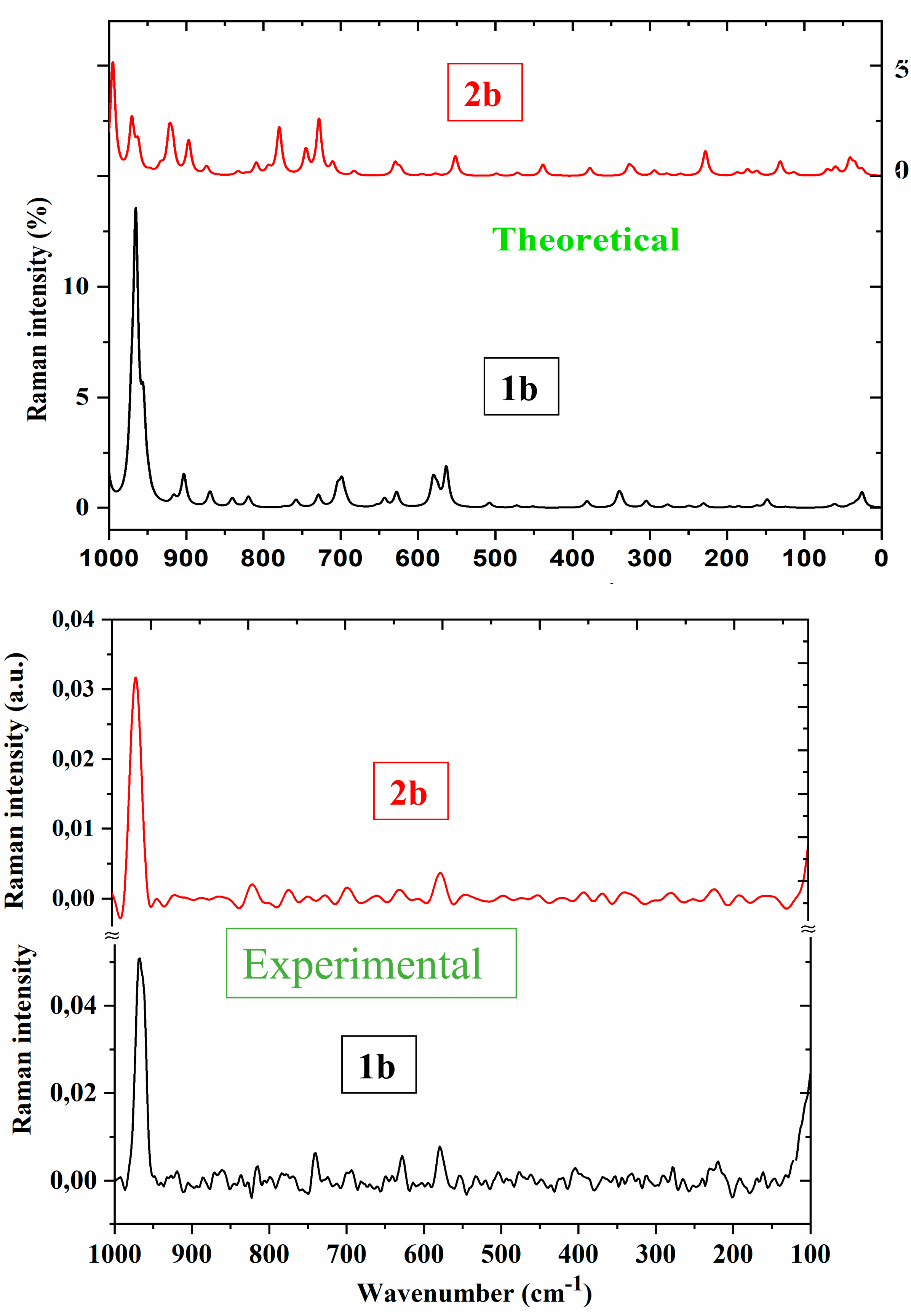

Figure S6. Comparison of the theoretical scaled Raman spectra at the B3LYP/6-31G(d,p) level with the experimental ones of **1b** and **2b** molecules in the 1000 - 0 and 1000 -100 cm^{-1} ranges, respectively.

Table S1. Calculated harmonic wavenumbers (ν, cm^{-1}), scaled (ν, cm^{-1}) wavenumbers obtained and the linear scaling equation procedure (LSE) and the polynomic scaling equation procedure (PSE), experimental (Exp) wavenumbers, relative infrared intensity (A) in % and relative Raman intensity (S) in % obtained at the B3LYP/6-31G(d,p) level in 1b and 2b molecules. In the main characterization of the different bands, the number of the ring mode corresponds to Wilson's notation [59].

	1b								2b							
	ν^{cal}	TLSE	PSE	A	S	Exp		Characterization	ν^{cal}	TLSE	PSE	A	S	Exp.		Characterization
No.		ν^{scal}	ν^{scal}			IR	Raman			ν^{scal}	ν^{scal}			IR	Raman	
1	3761	3606	3591	15	17	3516.0 w	3599.7vvw	ν(O-H) free (100)	-	--	--	--	--	--	--	-
	--	--	--	--	--	3420.6vw		ν(O-H) H-bonded	-	--	--	--	--	--	--	-
2	3246	3116	3114	0	6		3146.5 vw	2, ν(C3-H) in phenyl (100)	3240	3111	3109	0	7	3111.0 vw	3120.4 vw	2, ν(C3-H) in phenyl (100)
3	3241	3112	3110	0	6		3107.9 vw	20b,ν(C5-H) in phenyl (100)	3239	3110	3108	0	4	3103.3 vw	3111.8 vw	20b, ν(C5-H) in phenyl (100)
4	3220	3092	3090	0	8			7b, ν(C-H) in phenyl (100)	3207	3076	3075	2	11	3097.5 vw	3091.5 vw	7b, ν(C6-H) in phenyl (100)
5	3220	3092	3090	0	7		3084.8 vw	20a, ν(C-H) in phenyl (100)	3204	3073	3072	2	11			20a, ν(C2-H) in phenyl (100)
6	3173	3047	3046	2	5	3054.1w-m	3065.5 vw	ν_{as}(C-H) in $C_{18}H_2$ pyrrol (100)	3103	2980	2981	12	14		2986.4 vw	ν_{as}(C-H) in $C_{16}H_2$, $C_{17}H_2$ (100)
7	3123	2999	3000	7	11	2973.1 m	2990.3 vw	ν_{as}(C-H) in $C_{16}H_2$, $C_{17}H_2$ pyrrol	3089	2967	2968	8	13	2965.4 w	2965.2 vw	ν_{as}(C-H) in $C_{16}H_2$, $C_{17}H_2$ (100)
8	3115	2992	2992	5	14		2978.7 w	ν_{as}(C-H) in $C_{16}H_2$, $C_{17}H_2$ pyrrol	3078	2957	2958	19	11	2959.6 w	2953.6 vw	ν_{as}(C-H) in $C_{18}H_2$ pyrrol (100)
9	3078	2957	2958	5	5		2959.4 vw	ν(C-H) in $C_{15}H_2$, $C_{16}H_2$ pyrrol	3051	2931	2933	7	12	2941.3 vw	2935.3 vw	ν_s(C-H) in $C_{17}H_2$ pyrrol (100)
10	3063	2942	2944	4	6		2940.1 vw	ν_{as}(C-H) in $C_{16}H_2$, $C_{17}H_2$ pyrrol	3045	2925	2927	11	11	2926.8 vw	2918.9 vw	ν_s(C-H) in $C_{16}H_2$ pyrrol (100)
11	3057	2937	2938	3	10		2938.2 vw	ν_s(C-H) in pyrrol (100)	3018	2900	2902	5	4	2915.2 vw	2906.4 vw	ν_{as}(C-H) in $C_{15}H_2$ pyrrol (100)
12	3033	2914	2916	5	7		2911.2 vw	ν_s(C-H) in $C_{15}H_2$ pyrrol (100)	3001	2883	2886	6	6	2843.9 w	2880.3 vw	ν_s(C-H) in $C_{15}H_2$ pyrrol (100)
13	2986	2869	2872	11	9	2871.8 m	2866.8 vw	ν_s(C-H) in $C_{18}H_2$ pyrrol (100)	2937	2822	2826	22	15	2824.6 m	2810.9 vw	ν_s(C-H) in $C_{18}H_2$ pyrrol (100)
	--	--	--	--	--	2359.8 w	2847.6 vw	ν(O-H)$_{H\text{-}bonded}$, Combination band	--	--	--	--	--	2357.8 w	2362.5 vw	Combination band
						2342.4 w		ν(O-H)$_{H\text{-}bonded}$, Combination band						2339.8 w		Combination band
14	1800	1741	1749	85	16	1690.5vs	1644.2 w	ν(C=O) (91)	1762	1705	1713	75	3	1606.6 vs	1613.3 vw	ν_{as}(COO) (99)
15	1650	1599	1605	0	100		1594.0 vs	8a, ν(C=C) (100)	1652	1601	1607	24	100		1594.9 vs	8a, ν(C=C) (89)
16	1639	1588	1595	0	1			8b, ν(C=C) (98)	1624	1574	1581	4	0		1573.8 w	8b, ν(C=C) (82)
17	1609	1560	1566	100	20	1557.4 vs	1563.2 m	ν(C_8-N_{14}) (70) + ν_s(N7CC) (18)	1599	1550	1556	100	26	1543.9 m	1550.6 m	ν(C_8-N_{14}) (73) + ν_s(N7CC) (15)
18	1548	1502	1507	4	2		1520.7 vw	δ_s(C-H) in-phase in pyrrol (98)	1554	1507	1513	1	2		1513.9 w	δ(C-H) in-phase in pyrrol (92)
19	1541	1495	1501	30	32	1500.3 sh	1497.6 s	19a, ν(CC, CH) in phenyl (85)	1545	1499	1505	76	5	1497.6 s	1498.6 vs	ν(CN4)(37)+19a,ν(CC)(35)+δ(CH)pyrrol(15)
20	1536	1490	1496	3	2			δ_s(C-H) out-of-phase in pyrrol (97)	1540	1494	1500	20	2		1488.9vvw	δ_s(C-H) out-of-phase in pyrrol (88)
21	1514	1469	1475	15	3			δ(C-H) out-of-phase in pyrrol (75)	1514	1469	1475	1	4	1482.2 w	1485.1 w	δ_s(C-H) out-of-phase in pyrrol (85)
22	1512	1467	1473	25	6	1481.2vs		δ(C-H) (40) + ν(C-C_{11}) (35)	1510	1466	1471	10	11	1471.6 m	1471.6 m	ν_s(CN)in triazol(42)+δ_s(C-H)in pyrrol (35)
23	1504	1460	1465	2	2	1462.0 m	1451.3 vw	δ_s(C-H) out-of-phase in pyrrol (98)	1501	1457	1462	0	2	1462.9 m	1466.7 w	δ_s(C-H) out-of-phase in pyrrol (99)
	--	--	--	--	--	--	--	--	--	--	--	--	--	--	--	--
24	1460	1418	1423	2	0	1419.5 w	1434.0 vw	19b, ν(CC, CH) in phenyl (72)	1457	1415	1420	0	1	1417.6 vw	1427.2 vw	19b, ν(CC, CH) in phenyl (97)
25	1422	1382	1386	1	29		1412.7 vw	ν_{as}(NNN)(45)+ν(CN)(25)+δ(CH)(20)	1436	1395	1400	0	10	1391.6 w	1395.4 s	19a,ν(CC,CH)phenyl(40)+ν(C4N)(32)
26	1406	1367	1371	18	43	1378.1 m	1379.9 vs	ν(C4N)(35)+ν(NNN)(30)+δ(CH)(25)	1400	1361	1365	29	5	1369.4 m	1373.2 vs	γ_s(C-H) in-phase in pyrrol (93)
27	1392	1353	1357	7	3			γ(C_{18}H) in pyrrol (60)+ν(N_{10}C)(25)	1395	1356	1360	10	1	1340.5 m	1345.2 w	ν_s(NNN)(45)+γ_s(CC,CH) in pyrrol (35)
28	1386	1348	1351	9	5	1349.1 w	1347.2 w	γ(C-H) in pyrrol mainly in $C_{15}H_2$ (75)	1358	1321	1324	5	1		1324.0 vw	14,ν(CC) in phenyl(42)+γ(CH,CC)pyrrol(33)
29	1364	1327	1330	0	2	1332.7 w		14,ν(CC) in phenyl (50)+ν_{as}(NNN)(30)	1354	1317	1320	3	2		1318.2 vw	ν_{as}(NNN)(36)+γ(CH) in pyrrol (30)

30	1358	1321	1324	0	1			γ(C-H) in pyrrol (96)	1348	1312	1315	9	1		1314.4vvw	γ(C-H) in pyrrol (73)
31	1341	1305	1308	6	9		1306.7 vw	γ(CH) in pyrrol(40)+ν(CN)(30)+δ(OH)(20)	1340	1304	1307	46	8	1301.9 m	1306.7 w	ν_s(COO)(63)+γ_{as}(CH)pyrrol(18)+ν(N7C)(15)
32	1329	1293	1296	2	3	1297.1 w	1291.2 vw	Γ(C-H) in pyrrol (92)	1326	1291	1293	9	1	1290.3 w	1292.2 w	Γ(C-H) in pyrrol (94)
33	1326	1291	1293	0	2			3, δ(C-H) in phenyl (82)	1320	1285	1288	1	0	1277.8 m	1280.6 w	3, δ(C-H) in phenyl (97)
34	1302	1268	1270	0	3	1269.1 m	1268.1 w	ν_{as}(NN)(65)+3,δ(CH) in phenyl (25)	1284	1251	1253	16	3	1244.0 vw	1268.1 vw	γ_{as}(C-H) in pyrrol (98)
35	1281	1248	1250	3	2			γ_{as}(C-H) in pyrrol (93)	1258	1226	1228	3	8	1217.0 w	1232.4 w	ν(NN,CN)(68)+γ_{as}(CC,CH) in pyrrol (19)
36	1269	1236	1238	10	2	1241.1 m	1243.0 vw	δ(OH)(40)+ν(CN)(30)+γ_{as}(CH) in pyrrol(20)	1243	1212	1213	0	2		1213.1 vw	γ_{as}(C-H) out-of-phase in pyrrol (96)
37	1256	1224	1226	3	1		1212.2 vw	γ_{as}(C-H) out-of-phase in pyrrol (93)	1230	1199	1201	4	1		1200.6 vw	ν_{as}(NN,CN)(55)+3,δ(CH) in phenyl(22)
38	1215	1185	1186	2	0	1197.7 m	1196.7 vw	γ_{as}(C-H) out-of-phase in pyrrol (88)	1212	1182	1183	9	0	1182.3 vw	1192.9 vw	ν_{as}(NN,CN)(48)+ν(CN,CC) in pyrrol(35)
	--	--	--	--	--	--	--	--	--	--	--	--	--	--	--	--
39	1207	1177	1178	5	1		1179.4 vw	γ(C-H) in pyrrol (88)	1201	1172	1172	4	2	1164.8 vw	1169.7 m	γ_{as}(C-H) out-of-phase in pyrrol (83)
40	1198	1169	1170	0	7	1163.0 m	1167.8 m	9a, δ(C-H) in phenyl (80)	1189	1160	1161	3	13	1161.1 vw	1165.5 m	9a, δ(C-H) in phenyl (93)
41	1186	1157	1158	3	2	1148.5 m	1133.1 w	γ_{as}(C-H) in pyrrol (81)	1186	1157	1158	3	1		1158.2 vw	γ_{as}(C-H) out-of-phase in pyrrol (91)
42	1153	1126	1126	13	0		1125.4 w	δ(C-H) in pyrrol (92)	1151	1124	1124	1	0	1111.9 vw	1119.6 w	δ(C-H) in pyrrol (93)
43	1139	1113	1112	37	1	1112.9vw	1117.7 vw	ν_{as}(COO)(38)+δ(CH)(25)+δ(OH)(17)	--	--	--	--	--	--	--	--
44	1133	1107	1107	4	0		1102.3 vw	15, δ(C-H) in phenyl (94)	1124	1099	1098	1	0	1099.4 w	1097.4 vw	15, δ(C-H) in phenyl (99)
45	1117	1092	1091	15	1			ν(NNN)(38)+ν_s(COO)(30)+18a,δ(CH)(25)	1099	1075	1074	2	0		1079.1 vw	ν_s(NNN)(48)+18a,δ(C-H) in phenyl(42)
46	1108	1083	1082	13	7	1088.8 s	1090.7 m	ν(C-CL) (60) +18a,δ(CC) in phenyl (40)	1097	1073	1072	2	14	1089.7 m	1089.7 s	ν(C-CL)(32) +1,δ(CC) in phenyl(30)+ν_{as}(NNN)(28)
	--	--	--	--	--	--	--	--	--	--	--	--	--	--	1051.1vw	--
47	1061	1039	1037	0	1			γ_s(C-H) in pyrrol (80)	1055	1033	1031	1	1		1028.0 w	ν(CC) in pyrrol (72)
48	1029	1008	1006	2	4	1012.6 m	1011.6 w	12, δ(CC, CH) in phenyl (90)	1018	998	995	1	4	1008.7 w	1013.5 m	12, δ(CC, CH) in phenyl (96)
49	993	974	971	2	2		992.3 w	γ(C-H) in pyrrol (83)	993	978	971	1	2		970.1 vs	δ(CC,CH) in pyrrol (73)
50	988	973	966	15	10	968.2 m	967.3 s	ν_s(NNN)(43)+12,δ(CC)(25)+ν_s(CN14C)(20)	985	970	963	4	1		956.6 vw	γ_{as}(C-H) in pyrrol (87)
51	978	963	956	2	3		948.0 vw	ν_s(NNN)(50)+γ(C-H) in pyrrol (35)	969	954	947	0	0	950.2 w	943.1 vw	17a, γ(C-H) in phenyl (98)
52	976	961	954	0	0			17a, γ(C-H) in phenyl (99)	954	939	933	0	0		935.4 vvw	5, γ(C-H) in phenyl (99)
53	970	955	948	0	0	937.4 vw	938.2 vw	5, γ(C-H) in phenyl (100)	943	928	922	26	1	970.1 vs	923.9 vw	ν_s(NNN)(60)+ν(C8C)(25)+ν(CC)(15)
54	937	922	916	1	0	916.1 w	919.0 w	γ_{as}(C-H) in pyrrol (100)	939	924	918	18	1	909.4 vw	918.1 vw	γ_{as}(C-H) in pyrrol (86)
55	924	909	903	0	1		886.3 vw	γ_s(C-H) in pyrrol (100)	917	902	897	1	1		899.8 vw	γ_s(C-H) in pyrrol (98)
56	889	874	869	0	1	--	870.8 m	γ_{as}(C-H) in pyrrol (98)	893	878	873	1	0		875.6 vw	γ_{as}(C-H) in pyrrol (99)
57	859	844	840	0	0		861.2 w	γ_{as}(C-H) in-phase in pyrrol (97)	852	837	833	1	0	826.5 vs	830.3 vw	γ_{as}(C-H) in pyrrol (97)
58	849	834	830	8	0	825.5 s	836.1 vw	17b, γ(C-H) in phenyl (100)	841	826	823	7	0	814.9 vs	821.7 w	17b, γ(C-H) in phenyl (99)
59	838	823	820	0	0		814.9 w	10a, γ(C-H) in phenyl (100)	828	813	810	0	0	808.1 sh	813.9 w	10a, γ(C-H) in phenyl (100)
	--	--	--	--	--	--	--	--	811	796	793	2	0		789.8 vw	γ_s(COO) (54) + γ_s(CC9C) (35)
60	789	774	772	4	0	785.0 m	784.0 w	γ(C8C) (45) + γ(OCO) (30)	797	782	780	17	2	782.1 vvw	767.7 vw	δ_{as}(COO) (57) + γ(CC) (32)
61	775	760	758	1	0	768.6 vw	768.6 vw	6a,δ(CC) in phenyl (42)+δ(CC)pyrrol(28)	762	747	745	8	1	754.1 w	752.2 w	δ(CC)(55)+δ_s(COO)(28)+δ(triazole)(15)
62	745	730	729	1	0	733.9 m	724.3 vw	δ(CC) pyrrol (53)+6a,δ(CC) in phenyl(25)	744	729	728	0	2		722.3 vw	6a,δ(CC) in phenyl(42)+δ(CC) pyrrol (28)
63	720	705	704	6	1	704.9 vw		γ(C=O)(38)+γ(C8CN)(30)+γ(OH)(25)	726	711	710	0	0		710.8 vw	γ(C8C) (45) + γ_s(OCO) (36)
64	714	699	699	12	1	747.4 m*	739.7 m*	δ(COOH) (45) + δ(NNN) (34)	697	682	682	0	0	658.7 w	697.3 w	4, γ(CC) in phenyl (98)
65	708	693	693	0	0	696.3 vw	693.4 w	4, γ(CC) in phenyl (100)	--	--	--	--	--	--	--	--
66	668	653	654	4	0	661.5 w	649.0 vw	γ_s(NNN) (70)	644	629	630	0	0	641.3 w	629.7 w	6b, δ(CC) in phenyl (86)
67	657	642	643	4	0	648.0 w	641.3 vw	δ_{as}(NNN) (35) + 6b, δ(CC) (28)	637	622	623	3	0	627.8 vw	612.4 vw	γ_{as}(NNN) (58) + γ_s(OCO) (22)

68	641	626	627	0	1	627.8 vw	627.8 w	6b, δ(CC) in phenyl (73)	608	593	595	4	0	603.7 vw	594.1 vvw	γ_s(NNN) (63) + γ(CC8N) (18)
69	593	578	580	9	1		932.5vw*,579.6m	γ(O-H) (82)	--	--	--	--	--		--	--
70	588	573	575	3	0	--		δ_s(CC) pyrrol (82)	590	575	577	0	0		574.8 w	δ_{as}(C-H) in pyrrol (87)
71	576	561	563	5	1		554.6 w	δ(CC) in pyrrol (32)+δ(NNN)(30)	564	549	552	5	1		555.5 w	ν(CCL)(38)+δ(NNN)(32)+6b,δ(CC)(15)
72	519	503	508	2	0		504.4 vw	16b, γ(CC) in phenyl (91)	510	494	499	1	0		491.9 vw	16b, γ(CC) in phenyl (91)
73	483	467	472	0	0		477. 4 vw	δ(CC) in phenyl(34) +δ(NNN)(30)+δ(CO)(28)	482	466	471	1	0		472.6 vw	δ(CC)phenyl(35)+δ(NNN)(28)+δ(COO)(22)
74	462	446	452	4	0		462.0 vw	δ(CC) in phenyl(45) +δ(NNN)(28)+δ(CO)(25)	449	433	439	3	0		446.6 vw	δ(CCL)(32)+δ(CC)(25)+δ(NNN)(22)+δ(COO)(20)
--	--	--	--	--	--		--	--	--	--	--	--	--		--	
75	421	405	411	0	0		404.1 w	16a, γ(CC) in phenyl (100)	426	410	416	0	0		412.8 vw	10a, γ(CC) in phenyl (100)
76	390	374	381	0	0		369.4 vw		387	371	378	1	0		387.7 vw	
77	349	333	340	0	0		352.1 vw		335	319	327	0	0		345.3 vw	
78	345	329	337	0	0		330.8 vw		330	314	322	0	0		325.1 vw	
--	--	--	--	--	--		--		--	--	--	--	--		--	
79	313	297	305				311.6 vw		302	286	294	0	0		298.1 vvw	
80	285	269	277				278.8 w		286	270	278	0	0		277.8 vw	
81	257	241	250				265.3 vw		268	252	261	1	0		247.9 vw	
82	237	221	230				230.6 w		235	219	228	1	1		220.0 vw	
83	203	187	197				219.0 w		193	177	187	1	0		185.2 vvw	
84	191	175	185				192.0 vw		180	164	174	0	0		178.5 vvw	
85	168	152	162				161.1 vw		168	152	162	1	0		157.3 vw	
86	154	138	148				145.7 vw		137	121	131	0	0		129.3 vw	
87	130	114	124				132.2 vw		119	103	114	1	0		118.7 vw	
88	73	57	68						75	59	70	0	0			
89	66	50	61						65	49	60	0	0			
90	46	30	41						62	46	57	0	0			
91	43	27	38						46	30	41	0	0			
92	37	21	32						39	23	34	0	0			
93	30	14	26						30	14	26	0	0			

*Assigned to the dimer form. †Observed frequencies characterized by notation: vs= very strong, s= strong, m= medium, ms= medium strong, w=weak band, vw= very weak, vvw= very very weak, sh
shoulder. ν_{as}: anti-symmetric stretching, ν_s: symmetric stretching, δ: in-plane bending, γ: out-of-plane bending, β: scissoring, ρ: rocking, ω: wagging, τ: torsion

Table S2. Scaled ^1H NMR Chemical Shifts in **1b** with different solvents.

solvent	CH_2 H16'	CH_2 H16	CH_2 H17'	CH_2 H17	CH_2 H15'	CH_2 H15	CH_{Ar} H6	CH_{Ar} H2	CH_{Ar} H3	CH_{Ar} H5
Methanol	2.16	2.08	2.08	2.08	3.39	3.66	7.39	7.39	7.95	8.04
DMSO	2.10	2.02	2.02	2.02	3.35	3.62	7.40	7.40	7.97	8.06
1,4-dioxane	2.13	2.02	2.18	2.06	3.42	3.70	7.36	7.36	7.98	8.11
